# Effects of tyrosine kinase inhibitors on skeletal muscle metabolism: a link with muscle complaints

**DOI:** 10.1007/s00421-026-06287-6

**Published:** 2026-06-06

**Authors:** Lando Janssen, Charlotte A. Hoogstraten, Neeltje A. E. Allard, Tom J. J. Schirris, Maria T. E. Hopman, Nicole M. A. Blijlevens, Silvie Timmers

**Affiliations:** 1 Radboud Institute for Health Sciences, Department of Hematology, Radboud University Medical Center, Nijmegen, The Netherlands; 2 Radboud Institute for Health Sciences, Department of Physiology, Radboud University Medical Center, Nijmegen, The Netherlands; 3 Radboud Institute for Molecular Life Sciences, Department of Pharmacology and Toxicology, Radboud Centre for Mitochondrial Medicine, Radboud University Medical Center, Nijmegen, The Netherlands

**Keywords:** Chronic myeloid leukemia, Tyrosine kinase inhibitors, Muscle complaints, Mitochondrial function, Energy metabolism

## Abstract

Chronic myeloid leukemia (CML) patients receiving tyrosine kinase inhibitors (TKIs) often report muscle complaints (MC). Strategies to prevent or attenuate these muscle complaints remain enigmatic as the underlying mechanism is unclear, although a central role for mitochondria is proposed. We investigated the effects of TKIs on cellular metabolism in muscle biopsies of CML patients (*n* = 20), compared with healthy controls (*n* = 10) and whether these changes may be linked to muscle complaints. C2C12 myotubes were used to further explore the effects of TKIs on muscle metabolism *in vitro.* We found that TKI muscle concentrations in CML patients aligned with the concentrations at which cytotoxicity could be observed in C2C12 myotubes, and that muscle to plasma ratios tended to correlate with the degree of MC in CML patients. In C2C12 myotubes, acute TKI incubation of C2C12 myotubes resulted in alterations in energy metabolism, showing a shift from glycolysis to oxidative phosphorylation. CML patients without MC showed higher oxidative capacity, characterized by higher mitochondrial complex activities in skeletal muscle and occurrence of the first ventilatory threshold at a higher percentage of peak oxygen uptake during maximal exercise compared to patients with MC. Taken together, these observations raise the possibility that TKIs may affect cellular energy metabolism and may contribute to muscle complaints.

*Trial registration* The Netherlands Trial Registry identifier NTR6373.

## Introduction

The introduction of tyrosine kinase inhibitors (TKIs) in the early 2000s has remarkably improved the prognosis of patients with Philadelphia chromosome-positive chronic myeloid leukemia (CML) (Hochhaus et al. 2009). Consequently, the prevalence of CML has increased over the last two decades and is not expected to reach a plateau level before 2050 (Huang et al. 2012). Although TKIs are generally well-tolerated, muscle complaints (MC) such as pain and cramps are frequently reported (Jabbour et al. 2011). Patients experiencing muscle cramps have significantly lower TKI adherence, contributing to diminished disease control (Marin et al. 2010). In addition, muscle complaints are highly correlated with fatigue, which is the major factor limiting health-related quality of life in CML patients receiving TKI treatment (Efficace et al. 2013, Janssen et al. 2020). Previously, we found changes in muscle contractile properties in CML patients + MC, including a lower maximal rate of force rise and a trend toward prolonged relaxation time, while maximal exercise performance, assessed using a cycle ergometer test, was not impaired (Janssen et al. 2019). Nevertheless, no effects of TKI therapy on skeletal muscle mitochondrial density and ATP production capacity were found (Janssen et al. 2019). Yet, alterations in cellular metabolism are suggested as a plausible mechanism underlying these TKI-induced MC and the associated fatigue. Therefore, this study set out to explore in detail the effects of TKIs on cellular metabolism and to investigate potential connections to muscle related side effects reported among CML patients receiving TKI therapy. To this end, we measured muscle mitochondrial metabolism markers to investigate cellular changes at rest, and assessed energy metabolism during maximal exercise to explore how these alterations may manifest under physiological stress in CML patients with and without MC. We assessed TKI plasma trough and muscle concentrations, and compared those levels with a cytotoxicity assay using the same drugs in C2C12 myotubes. Additionally, we measured oxygen consumption and extracellular acidification rate in C2C12 myotubes after TKI treatment. These analyses aimed to gain insight into how cellular metabolism responds to near toxic concentrations of TKIs and to support the observations obtained from patient muscle samples. We hypothesized that treatment with TKIs would induce a reduction in mitochondrial function, both at rest and during maximal exercise, and that these changes would be more pronounced in CML patients reporting muscle complaints. Furthermore, we expected that TKI concentrations approaching cytotoxic levels in vitro would correlate with mitochondrial and metabolic alterations observed in patient muscle samples, providing a mechanistic link between TKI exposure and muscle-related side effects.

## Methods

### Human data

#### Determination of OXPHOS complex capacity and TKI concentrations in patient muscle biopsies and plasma

Muscle biopsies and plasma were used from 20 CML patients receiving TKI treatment and 10 non-CML controls who participated in a previous study (Janssen et al. 2019), and subject characteristics are provided in Table 1. All subjects provided written informed consent as approved by the Local Committee on Research Involving Human Subjects of the region Arnhem and Nijmegen (The Netherlands Trial Registry identifier NTR6373). Mitochondrial complex activities were measured in fresh 600 *g* mitochondrial-enriched fractions (10% *w/v*) in SETH-buffer (0.25 M Sucrose, 2 mM EDTA, 10 mM Tris, 50 U/mL Heparin, pH 7.4) using spectrophotometry (Janssen et al. 2007). TKI concentrations in muscle biopsies and plasma were determined using high-performance liquid chromatography (HPLC) coupled with tandem mass spectrometry (MS/MS) (Erp et al. 2013). Plasma was drawn at least six hours after intake of the last dosage in order to estimate trough plasma concentrations using an established formula (Lankheet et al. 2017, Wang et al. 2009). To mimic the standard plasma matrix of this diagnostic HPLC-MS/MS method, muscle homogenates were diluted 1:5 (*v/v*) in EDTA plasma from a healthy control prior to measurement.

**Table 1:** Subject and hematological characteristics adapted from (Janssen et al. 2019)

Characteristic	CML patients	Controls	*P* value	CML + MC	CML – MC	*P* value
Subject number, N	20	10	–	10	10	–
Gender, male/female	14/6	7/3	1.00	7/3	7/3	1.00
Age, years	54 ± 8	58 ± 7	0.25	55 ± 9	54 ± 8	0.86
BMI, kg/m²	25.8 ± 4.1	27.5 ± 5.4	0.34	25.8 ± 3.6	25.8 ± 4.8	0.97
Physical activity, METmin/week; median (IQR)	2288 (1545–4982)	2850 (1965–4868)	0.53	2541 (1689–5488)	2070 (1403–4570)	0.63
Smoker, %	0	0	–	0	0	–
Age at Dx, years	46 ± 8	N/A	–	47 ± 7	46 ± 9	0.78
BCR-ABL level, N (%)	–	N/A	–			1.00
No MMR	1 (5)			1 (10)	0 (0)	
MMR or deeper	19 (95%)			9 (90)	10 (100)	
TKI, N (%)	Imatinib: 10 (50), Dasatinib: 5 (25), Nilotinib: 2 (10), Bosutinib: 2 (10), Ponatinib: 1 (5)	N/A	–	Imatinib: 6 (60), Dasatinib: 2 (20), Nilotinib: 1 (10), Bosutinib: 1 (10), Ponatinib: 0 (0)	Imatinib: 4 (40), Dasatinib: 3 (30), Nilotinib: 1 (10), Bosutinib: 1 (10), Ponatinib: 1 (10)	0.89
Duration of current TKI therapy, months; median (IQR)	42.5 (21.0–114.8)	N/A	–	66.5 (19.0–121.5)	38.0 (16.5–73.3)	0.63
Prior TKIs, N; median (IQR)	0.5 (0.0–1.0)	N/A	–	0.0 (0.0–1.0)	1.0 (0.0–1.0)	0.53

Values are presented as mean ± SD unless indicated otherwise. There were no significant differences in subject and hematological characteristics between CML patients, except for a higher Charslon Comorbidity Index score in CML patients Also, there were no significant differences between CML patients with and without TKI induced muscle complaints

*MC* muscle complaints, *BMI* body mass index, *MET* metabolic equivalent of task, *IQR* interquartile range, *Dx* diagnosis, *MMR* major molecular response, *TKI* tyrosine kinase inhibitor

#### Assessment of energy metabolism during maximal exercise in CML patients

An incremental maximal exercise test was performed in CML patients and controls according to the ACC/AHA guidelines, of which the maximal oxygen uptake have been published elsewhere (Janssen et al. 2019). The first ventilatory threshold (VT1) was determined, representing the point during incremental exercise where ventilation increases disproportionately to oxygen uptake, indicating a shift from predominantly aerobic toward increasing anaerobic metabolism (Beaver et al. 1986).

### In vitro data

#### Compounds

Imatinib, dasatinib, nilotinib, and bosutinib were obtained from Cayman Chemical Company. Asciminib was purchased from Selleck Chemicals LLC. The final DMSO concentration used to dissolve all TKIs equaled 0.1% (*v*/*v*).

#### Cell culture

C2C12 murine myoblasts were obtained from American Type Culture Collection (ATCC) and did not exceed 25 serial passages to exclude previously observed variability in C2C12 cells (Pronsato et al. 2013). Cells were maintained in Dulbecco’s modified Eagle’s medium (DMEM), containing 25 mM glucose, GlutaMAX™, and 25 mM HEPES (Gibco™ Life Technologies) supplemented with 10% (*v/v*) fetal bovine serum (Greiner Bio-One) at 37 °C in a humidified atmosphere of 5% (*v/v*) CO_2_. Myoblasts were seeded at a density of 4 · 10^4^ cells/cm^2^ to reach a plating density of 80–90% within 24 to 48 h. Subsequently, cells were differentiated into multinucleated myotubes by replacing medium with DMEM containing 5.5 mM glucose and 4 mM L-glutamine (Sigma-Aldrich) supplemented with 2% (*v/v*) horse serum (Sigma-Aldrich) for five days. Differentiation medium was changed every two days. Experimental data was normalized for cellular protein content, determined by a Bradford protein assay (Bio-Rad) after lysing cells with 1 M NaOH.

#### Cell-viability analysis

C2C12 cells were seeded in black/clear flat bottom 96-wells plates (Fisher Scientific). Multinucleated myotubes were exposed to TKIs in a serial √10 dilution (0.1–316 µM) for 30 min and 24 h. Nuclei were stained with hoechst 33,342 (20 µg/mL, Life Technologies), Yo-Pro-1-iodide (2 µM, Life Technologies) and propidium iodide (1 µg/mL, Sigma-Aldrich) for 30 min at 37 °C to differentiate all, early apoptotic, and necrotic cells, respectively (Schirris et al. 2015). Fluorescence images were acquired at a 100x magnification using a Becton Dickinson (BD) Pathway 855 high-throughput microscope (BD Bioscience). Images were analyzed for viable and non-viable cells using CellProfiler™ v3.0.0 (Schirris et al. 2015, Carpenter et al. 2006).

#### Determination of cellular respiration and extracellular acidification

Cellular oxygen consumption rates (OCR) and extracellular acidification rates (ECAR) were measured using the Seahorse XF96 extracellular flux analyzer (Agilent) to probe cellular metabolism. Cells were seeded in 96-wells Seahorse XF96 cell culture microplates (Agilent) and differentiated as described above. Differentiation medium was replaced one hour before initiation of the experiment by sodium bicarbonate-free DMEM containing 5.5 mM glucose, 1 mM sodium pyruvate, and 4 mM L-glutamine (Sigma Aldrich) supplemented with 20 mM HEPES, pH 7.4. For 24-hour TKI-exposures, TKIs were also added after replacement of the medium. Oligomycin (2.5 µM, Sigma Aldrich), FCCP (5 µM, Sigma Aldrich) and rotenone/antimycine A (Rot, 1 µM; AA, 2.5 µM, both Sigma Aldrich) were subsequently added to measure oxygen consumption rates (Nicholls et al. 2010). Next, OCR values were corrected for non-mitochondrial respiration (respiration after addition of Rot/AA) and for protein content. Subsequently, TKI pre-treated conditions were normalized to the baseline of the vehicle control samples. Conditions without TKI pre-treatment were normalized to their own baseline (first three OCR values). ECAR values were corrected for protein content and were normalized to the vehicle control at each timepoint. The last two time points before the following injection were used to compare OCR and ECAR levels between treatment conditions.

### Statistical analysis

Data is presented as mean ± SEM, unless stated otherwise. Mitochondrial complex activities in human biopsies were compared between groups using two-way ANOVA analysis after log-transformation, to correct for a skewed distribution of the data. Student’s t-tests and one-way ANOVA with Dunnett’s *post hoc* analysis were used to compare the effects of TKIs with vehicle control. Curve-fitting and statistical analysis was performed using GraphPad prism 5.03 software (GraphPad Software Inc.) and IBM SPSS Statistics 20.0.0.1 (IBM Corp.).

## Results

### TKIs may accumulate in human muscle tissue at concentrations associated with cytotoxic effects in C2C12 myotubes

TKI plasma trough and muscle concentrations in CML patients receiving TKI treatment with and without muscle complaints are displayed in Table 2. Comparison of the muscle TKI-concentration against their respective plasma concentration demonstrated slight muscle accumulation of imatinib, dasatinib, and bosutinib, which was not observed for nilotinib (Table 2). Among the TKIs, imatinib showed the highest muscle to plasma concentration ratios, aligning with its frequent clinical association with muscle complaints (Janssen et al. 2020). Ratios also showed a tendency to correlate with the severity of muscle complaints (*r* = 0.531, *p* = 0.057). To explore whether these concentrations could potentially affect skeletal muscle, we performed a cytotoxicity assay for the TKIs using C2C12 myotubes. TKI-concentrations ranging from 0.1 µM up to maximum solubility were used, and cytotoxicity was assessed in a dose-dependent manner after a 24-hour incubation. For dasatinib and bosutinib, cytotoxicity occurred at lower concentrations compared to imatinib and nilotinib (Fig. 1). Notably, the concentrations at which cytotoxicity was observed in C2C12 myotubes roughly correspond to the muscle TKI concentrations measured in CML patients (Fig. 1).

**Table 2:** Overview of TKI plasma trough and muscle concentrations in CML patients receiving TKI treatment with and without muscle complaints

Patient ID	TKI type	Muscle complaints	[TKI]_plasma trough_ (µg/L)^#^	[TKI]_muscle_ (µg/L)	[TKI]_muscle_ (µM)
1	Imatinib	Y	813.4	8 620	17.46
2	Imatinib	N	1 089	10 848	21.98
3	Imatinib	N	1 329	2 351	4.76
4	Imatinib	Y	1 439	2 894	5.86
5	Imatinib	Y	327.3	3 035	6.15
6	Imatinib	Y	867.8	11 298	22.89
7	Imatinib	Y	689.9	9 905	20.07
8	Imatinib	Y	1 175	8 363	16.97
9	Imatinib	N	1 555	10 072	20.41
10	Imatinib*	N	1 308	4 633	9.39
11	Dasatinib	N	–	54.13	0.11
12	Dasatinib	N	–	57.81	0.12
13	Dasatinib	Y	0.78	48.39	0.1
14	Dasatinib	Y	0.44	–	–
15	Dasatinib	N	2.43	60.84	0.12
16	Nilotinib*	N	1 857	1 390	2.62
17	Nilotinib*	Y	1 534	467.4	0.88
18	Bosutinib	N	57.55	2 635	4.97
19	Bosutinib	Y	–	3 561	7.3
20	Ponatinib	N	–	–	–

*Twice daily

#Plasma trough concentrations were calculated according to a previously published and established formula ADDIN EN.CITE (Lankheet et al. 2017, Wang et al. 2009)

**Fig. 1:**
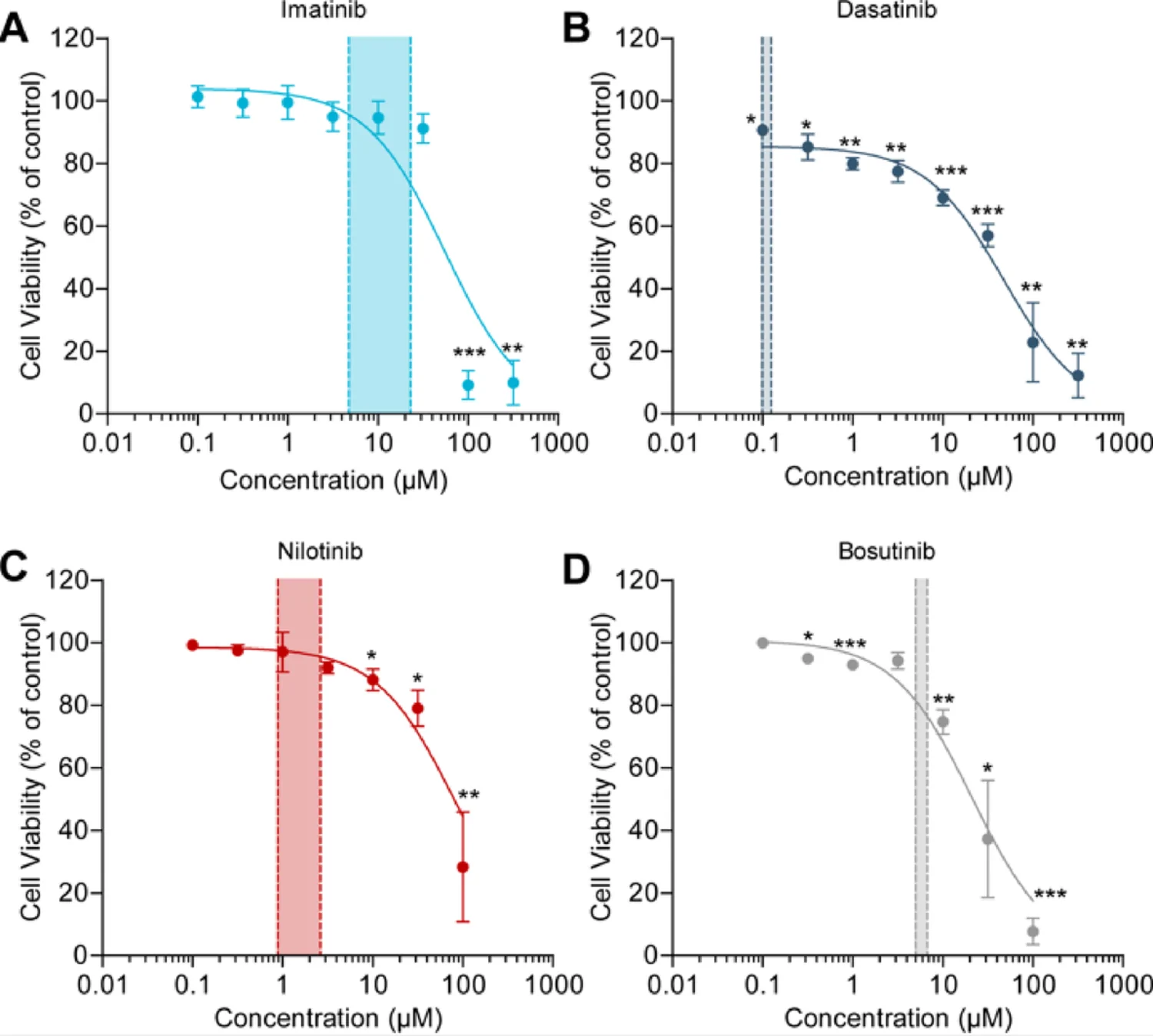
Dose-dependent effects on cytotoxicity in C2C12 myotubes upon 24 h TKI-incubation. Cell death was assessed in C2C12 myotubes after 24 h incubation with a serial √10 TKI dilution of (**A**) imatinib, **B** dasatinib, **C** nilotinib, and **D** bosutinib. All results were normalized to non-exposed controls. Statistical analyses: Student’s t-tests to compare differences in cell viability between vehicle control and TKI-exposed myotubes. *p < 0.05, ** *p* < 0.01, *** *P* < 0.001. Area between dotted lines represent the observed TKI concentrations in skeletal muscle of CML patients. Data is presented as mean ± SEM; *n* = 4 biologically independent experiments

### TKI-induced muscle complaints may be associated with lower skeletal muscle oxidative capacity

**Fig. 2:**
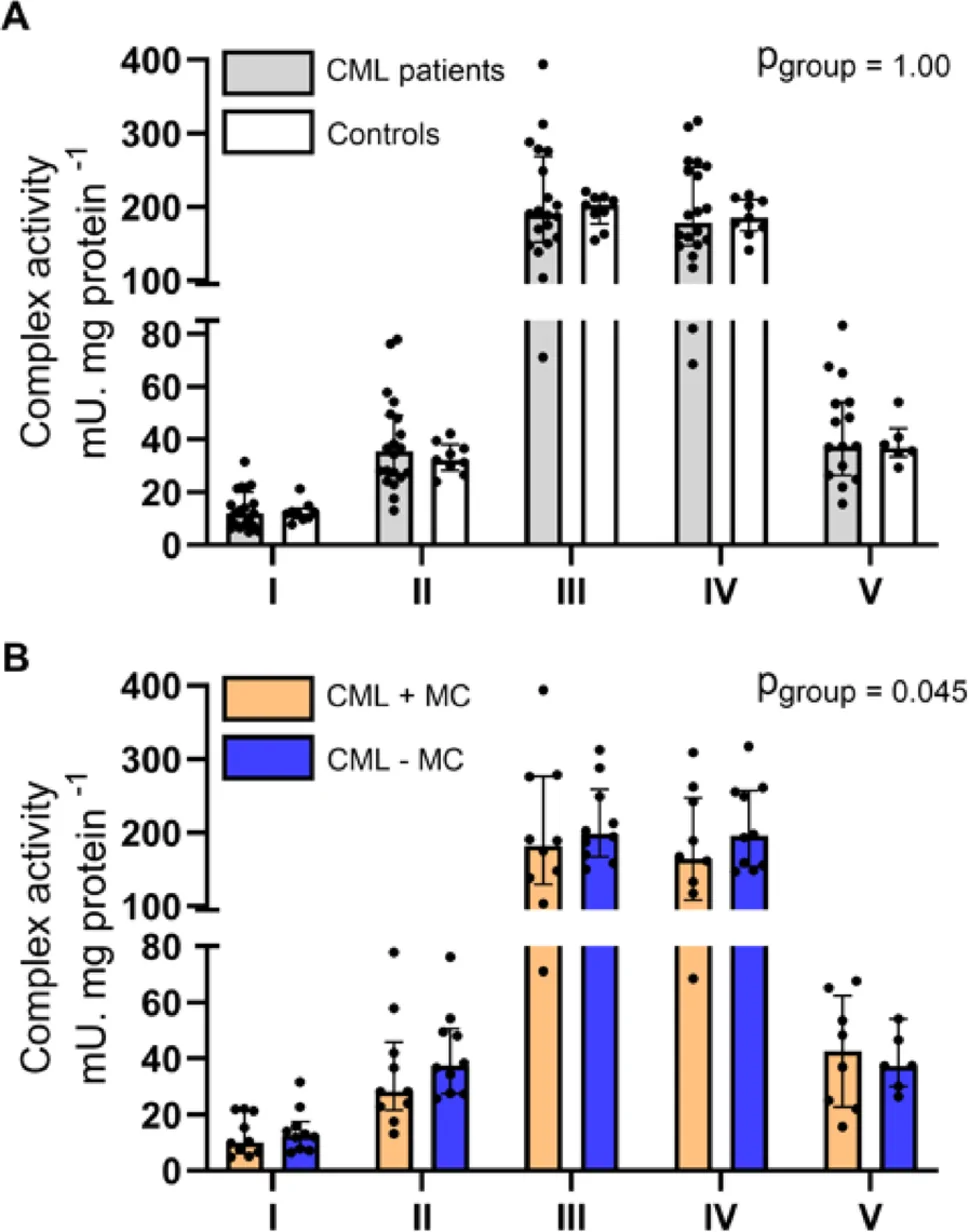
TKI effects on cellular metabolism in skeletal muscle biopsies from CML patients with and without muscle complaints. **A** Comparison of enzyme activity of individual OXPHOS complexes in skeletal muscle tissue between CML patients (*n* = 20) and non-CML controls (*n* = 10) and (**B**) between CML patients with muscle complaints (CML + MC, *n* = 10) and CML patients without muscle complaints (MCL-MC, *n* = 10). Non-log-transformed data are presented in the figure as scatter dot plots, with the line at the median with interquartile range. Statistical analyses: Mitochondrial complex activities were compared between groups (i.e., P_group_; considering all (CI - CV) OXPHOS complexes) using two-way ANOVA after log-transformation

No differences in mitochondrial complex activities at rest were found between CML patients and controls (Fig. 2A). Within CML patients, those with muscle complaints (CML + MC) exhibited lower complex activities compared to patients without muscle complaints (CML-MC, *p* < 0.05; Fig. 2B). During maximal exercise, the first ventilatory threshold (VT1) tended to occur at a lower percentage of peak oxygen uptake in CML + MC (58 ± 6%) compared to CML-MC (63 ± 7%; *P* = 0.08). While this difference did not reach statistical significance, it may indicate a possible reduction in oxidative capacity under physiological stress in patients with muscle complaints.

### TKIs may influence the balance between glycolysis end oxidative metabolism in C2C12 myotubes

**Fig. 3:**
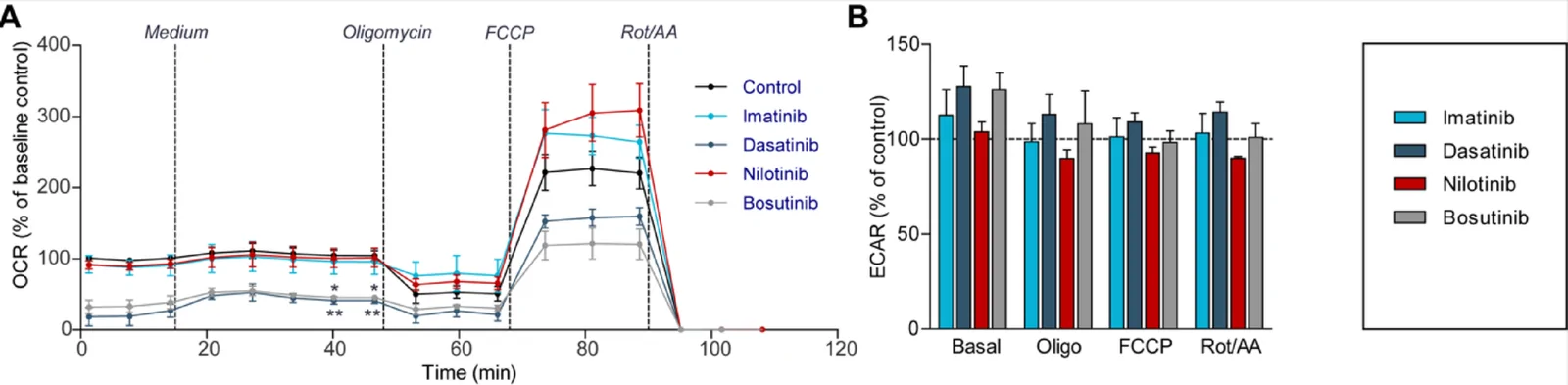
Cellular metabolic capacity in C2C12 myotubes upon 24 h TKI-incubation. Effects of TKIs on (**A**) oxygen consumption rates (OCR) and (**B**) extracellular acidification rates (ECAR) in C2C12 myotubes after a 24-hour incubation with 10 µM TKI. Statistical analyses: One-way ANOVA corrected for multiple comparison by Dunnett’s *post hoc* analysis was used for comparison of OCRs and ECARs. *p < 0.05, ** *p* < 0.01. Data is presented as mean ± SEM; *n* = 4 biologically independent experiments

To further explore the cellular mechanisms underlying our patient observations, we assessed the effects of TKIs on C2C12 myotube metabolism by measuring oxygen consumption rate (OCR) and extracellular acidification rate (ECAR). To this end C2C12 myotubes were incubated for 24 h with 10 µM of TKIs, approximating concentrations at which cytotoxic responses begin to appear (18). Dasatinib and bosutinib significantly lowered basal OCR compared to control, while maximal respiration (Fig. 3A) and ECAR (Fig. 3B) were not significantly affected. Notably, both TKIs exhibited cytotoxicity at low concentrations (Fig. 1), which could have contributed, at least in part, to the observed reduction in mitochondrial function.

**Fig. 4:**
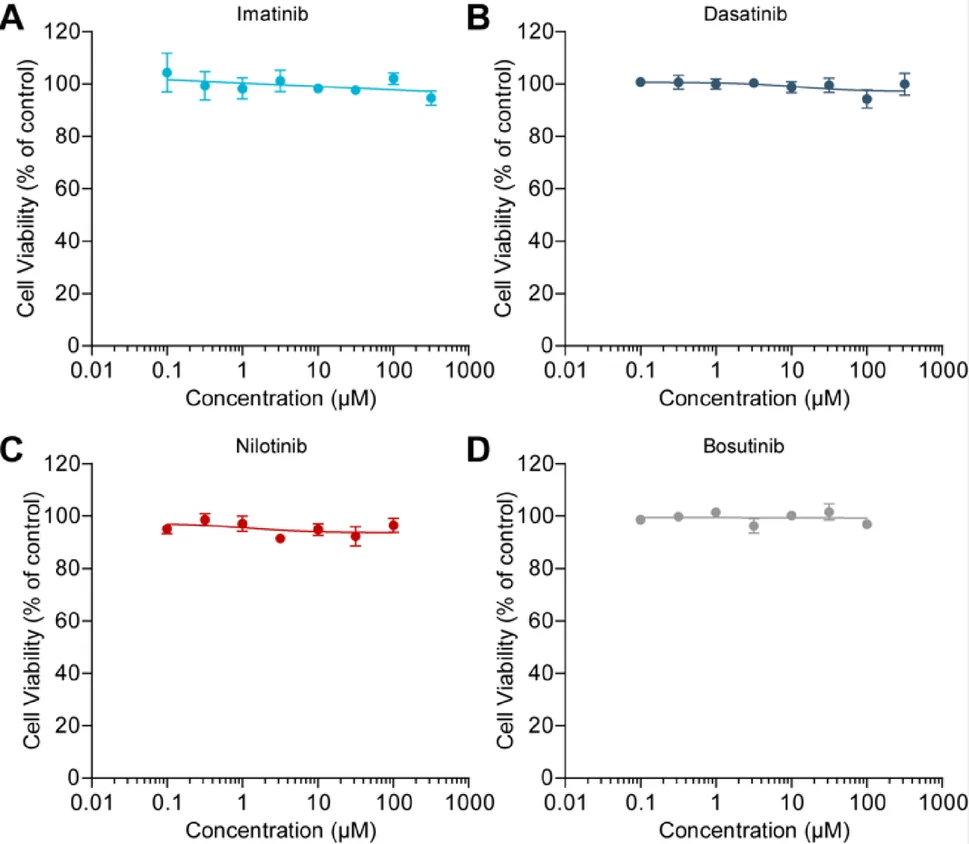
Dose-dependent effects on cytotoxicity in C2C12 myotubes upon 30 min TKI-incubation. Cell death was assessed in C2C12 myotubes after a 30-minute incubation with a serial √10 TKI dilution of (**A**) imatinib, **B** dasatinib, **C** nilotinib, and **D** bosutinib, normalized to non-exposed controls. Statistical analyses: Student’s t-tests to compare differences in cell viability between vehicle control and TKI-exposed myotubes. Data is presented as mean ± SEM; *n* = 4 biologically independent experiments

**Fig. 5:**
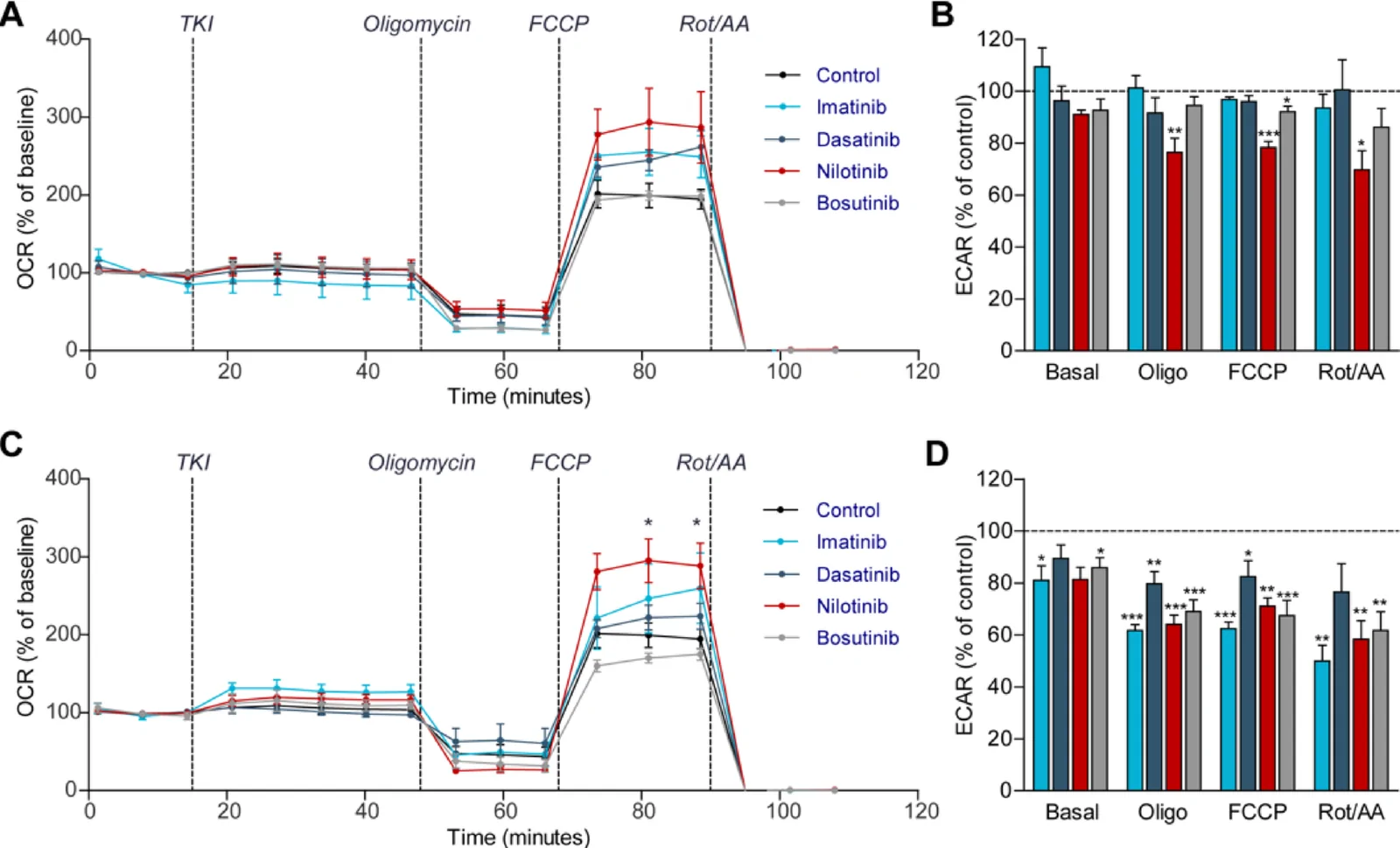
Cellular metabolic capacity in C2C12 myotubes upon 30 min TKI-incubation. OCR and ECAR were determined in C2C12 myotubes after a 30-minute incubation with 10 µM (**A**, **B**) and 100 µM (**C**, **D**) TKI. Statistical analyses: One-way ANOVA corrected for multiple comparison by Dunnett’s *post hoc* analysis was used for comparison of OCRs and ECARs. *p < 0.05, ** *p* < 0.01, *** *p* < 0.001. Data is presented as mean ± SEM; *n* = 4 biologically independent experiments

To minimize potential adaptive responses, cells were also exposed for 30 min to 10 µM and 100 µM TKIs. The higher dose was chosen based on data suggesting that concentrations up to 200-fold plasma C_max better predict TKI-induced toxicity in humans (19). Short-term incubation did not significantly affect cell viability (Fig. 4). Addition of 10 µM TKIs for 30 min did not alter OCR (Fig. 5A), but nilotinib significantly reduced ECAR during ATP-linked, maximal, and non-mitochondrial respiration (Fig. 5B). Direct addition of 100 µM TKIs increased OCR, reaching statistical significance for nilotinib (Fig. 5C), and augmented the reduction in ECAR for all TKIs (Fig. 5D).

These results suggest that TKIs may influence the balance between glycolytic and oxidative metabolism in C2C12 myotubes, with short-term and high-dose exposures highlighting potential shifts toward a more oxidative phenotype. However, some effects may be secondary to early cytotoxic stress, and further studies are needed to confirm these findings in vivo.

## Discussion

This study aimed to investigate the effects of TKIs on cellular metabolism and their potential link to muscle-related side effects in CML patients. Our exploratory analyses suggest that TKI concentrations in skeletal muscle tissue of CML patients correspond with concentrations at which cytotoxicity was observed in C2C12 myotubes and tend to correlate with the severity of muscle pain. Acute exposure of C2C12 myotubes to TKIs resulted in a shift towards a more oxidative phenotype. Consistent with this, CML patients without muscle complaints exhibited higher oxidative capacity, as reflected by increased mitochondrial complex activities and the occurrence of the first ventilatory threshold (VT1) at a higher percentage of peak oxygen uptake during maximal exercise compared to those with muscle complaints. These findings indicate that TKIs may influence skeletal muscle metabolism, and that reduced oxidative capacity could contribute to the presence of muscle complaints in CML patients.

The hypothesis that TKIs can induce metabolic alterations is not entirely novel. Previous studies have shown that TKIs can reverse the Warburg effect in CML cells by shifting metabolism from glycolysis toward oxidative metabolism (Vander Heiden et al. 2009). BCR-ABL-positive cells exhibited suppressed glycolytic activity and increased TCA cycle activity following imatinib exposure (Gottschalk et al. 2004, Kominsky et al. 2009). Similar effects of imatinib on glucose metabolism were reported in gastrointestinal stromal tumor cell lines (Vitiello et al. 2018), and more recent studies link metabolic alterations to TKI resistance in CML stem cells (Qiu et al. 2023). However, whether TKIs trigger metabolic changes in skeletal muscle, which could contribute to muscle complaints, remains poorly understood, especially at clinically relevant concentrations and in relation to cytotoxic effects. TKI concentrations in skeletal muscle have rarely been measured—apart from a recent study in bariatric surgery patients (Lau et al. 2024) —and have scarcely been linked to changes in skeletal muscle metabolism. Investigating this further is crucial to understand their potential contribution to muscle-related metabolic alterations.

C2C12 myotubes are widely used to study skeletal muscle metabolism due to their similarity to human muscle cells (Sarabia et al. 1990, Abdelmoez et al. 2020), and have previously been employed to investigate the effects of imatinib on energy metabolism (Damaraju et al. 2018). Our observations of cytotoxicity at concentrations corresponding to patient muscle levels suggest that TKIs may influence skeletal muscle metabolism beyond BCR-ABL-positive cells. Differences in mitochondrial complex activities and ventilatory thresholds between patients with and without muscle complaints suggest that TKI-induced muscle side effects may partially arise from alterations in cellular substrate metabolism.

The effects of TKIs on energy metabolism were more pronounced in C2C12 myotubes than in patient muscle tissue. This discrepancy may reflect the fact that biopsies were collected from patients in a fasted, unstressed state, whereas in vitro cells may be more sensitive to TKI exposure. Furthermore, human muscle maintains a more balanced energy production between fatty acid oxidation and glycolysis, while C2C12 cells primarily rely on glycolysis (Abdelmoez et al. 2020).

In contrast to our findings, previous studies have reported inhibitory effects of short-term TKI exposure on mitochondrial complexes, including inhibition of complex V by imatinib (Damaraju et al. 2018, Will et al. 2008) and of complexes IV and V by dasatinib (Will et al. 2008). However, these studies were conducted in isolated and disrupted mitochondria, which may differences compared to intact cell measurements.

Several study limitations should be noted. The small sample size and inclusion of patients on different TKIs necessitate cautious interpretation. Nonetheless, to our knowledge, this is the first study providing insight into skeletal muscle TKI concentrations in CML patients. Furthermore, the findings of the present study remain largely descriptive, and the in vitro experiments in C2C12 myotubes were not designed to fully elucidate the underlying mechanisms. Future studies are needed to further explore the mechanistic links between TKI treatment, alterations in muscle metabolism, and the development of muscle complaints. Our findings merely provide a foundation for further investigation of the mechanisms by which TKI therapy affects skeletal muscle and contributes to muscle complaints.

In conclusion, our data suggest that TKIs may influence cellular energy metabolism in skeletal muscle. These metabolic alterations could contribute to TKI-induced muscle complaints in CML patients. While our study provides initial insights, further research is needed to delineate the precise mechanisms linking TKI exposure to alterations in skeletal muscle metabolism and the development of muscle complaints. Such studies could inform future strategies to prevent or mitigate these side effects in patients undergoing TKI therapy.

## Data Availability

The datasets generated during and/or analysed during the current study are available from the corresponding author on reasonable request.

## Abbreviations

CML: Chronic myeloid leukemia
MC: Muscle complaints
TKI: Tyrosine kinase inhibitors
HPLC: High performance liquid chromatography
MS/MS: Tandem mass spectrometry
VT1: First ventilatory threshold
ATCC: American type tissue culture
DMEM: Dulbecco’s modified eagle’s medium
OCR: Cellular oxygen consumption rates
ECAR: Extracellular acidification rates
BMI: Body mass index
MET: Metabolic equivalent of task
IQR: Interquartile range
Dx: Diagnosis
